# Adherence to inhaled therapy and its impact on chronic obstructive pulmonary disease (COPD)

**DOI:** 10.1186/s12890-018-0724-3

**Published:** 2018-10-19

**Authors:** Magdalena Humenberger, Andreas Horner, Anna Labek, Bernhard Kaiser, Rupert Frechinger, Constanze Brock, Petra Lichtenberger, Bernd Lamprecht

**Affiliations:** 1 Department of Pulmonology, Kepler University Hospital, Krankenhausstrasse 9, A4021, Linz, Austria; 2 Faculty of Medicine, Johannes-Kepler-University, Linz, Austria; 3 Department of Health Economics, Upper Austrian Health Insurance, Linz, Austria; 4 Department of Medical Controlling, Kepler University Hospital, Linz, Austria

**Keywords:** Chronic obstructive pulmonary disease, Adherence, Inhaled therapy

## Abstract

**Background:**

COPD is a treatable disease with increasing prevalence worldwide. Treatment aims to stop disease progression, to improve quality of life, and to reduce exacerbations. We aimed to evaluate the association of the stage of COPD on adherence to inhaled therapy and the relationship between adherence and COPD exacerbations.

**Methods:**

A retrospective analysis of patients hospitalized for acute exacerbation of COPD in a tertiary care hospital in Upper Austria and discharged with a guideline conform inhaled therapy was performed. Follow-up data on medical utilization was recorded for the subsequent 24 months. Adherence to inhaled therapy was defined according to the percentage of prescribed inhalers dispensed to the patient and classified as complete (> 80%), partial (50–80%) or low (< 50%).

**Results:**

Out of 357 patients, 65.8% were male with a mean age of 66.5 years and a mean FEV_1_ of 55.0%pred. Overall, 35.3% were current smokers, and only 3.9% were never-smokers. In 77.0% inhaled triple therapy (LAMA + LABA + ICS) was prescribed. 33.6% showed complete adherence to their therapy (33.2% in men, 34.4% in women), with a mean age of 67.0 years. Mean medication possession ratio by GOLD spirometry class I – IV were 0.486, 0.534, 0.609 and 0.755, respectively (*p* = 0.002). Hence, subjects with complete adherence to therapy had a significantly lower FEV_1_ compared to those with low adherence (49.2%pred. vs 59.2%pred., respectively; *p* <  0.001).

The risk of exacerbations leading to hospitalization was 10-fold higher in GOLD spirometry class IV compared to GOLD spirometry class I, which was even more evident in multivariate analysis (OR 13.62).

**Conclusion:**

Complete adherence to inhaled therapy was only seen in 33.6% and was higher among those with more severe COPD.

**Trial registration:**

Not applicable.

## Background

Chronic obstructive pulmonary disease (COPD) is an underdiagnosed, preventable and treatable disease with increasing prevalence worldwide. It has been a major problem over decades and will be a challenge within the twenty-first century [1–4].

To reduce mortality in COPD patients, lower the economic and clinical burden and to improve quality of life, it is crucial to prevent disease progression, reduce exacerbation rates and focus on the treatment of comorbidities [5–9]. Adherence to inhaled therapy appears to have significant impact on treatment goals. Therefore, it is crucial to increase the patients’ and physicians’ awareness concerning this topic.

Only few data are available on adherence and influencing factors. A meta-analysis of over 50 years of research on adherence shows an association between adherence and social and emotional resources [10, 11]. In a study of patients including those with COPD by Balkrishnan et al., the numbers of hospitalization rates and physician visits were reduced in patients who were adherent to prescribed therapy [12].

Nonadherence is a tremendous problem in the treatment of patients in general [13]. Furthermore, adherence in COPD patients is particularly poor and reported nonadherence rates range from 50 to 80% [14–16].

In patients with COPD, nonadherence to inhaled therapy is caused by several factors and could lead to high mortality and morbidity as well as hospitalizations and a reduced quality of life [14, 17–21]. Thus, the consequences of nonadherence, clinically and economically, are neither completely obvious nor fully understood, but there is an association between nonadherence and increasing healthcare costs [17, 19, 22, 23].

The aim of this retrospective data analysis was to evaluate the association of the stage of COPD on adherence and the relationship between adherence and COPD exacerbations. We hypothesized, that better adherence is associated with less COPD exacerbations leading to hospitalization.

## Methods

The primary outcome parameter of this retrospective analysis was to describe the characteristics of an Upper Austrian COPD cohort based upon degree of adherence to inhaled therapy and its association with spirometrically defined COPD stages. Moreover, we explored adherence as a risk factor for the poor outcome of exacerbation risk and we described further influencing factors on adherence.

Data of patients hospitalized for COPD exacerbations at the department for pulmonology in a tertiary care hospital in Upper Austria and discharged with a guideline conform inhaled therapy in 2012 were analyzed. The following observation period was 24 months. Patients who died within the first six months of the observation period were excluded due to the short observation period. However, patients who died afterwards but during the observation period, were included until death. Hence, the observation period was shorter in these patients and it was assumed that these individuals would have continued with the same adherence routine prior to their death.

Inclusion criteria were age > 40 years, COPD diagnosis (GOLD spirometry class I – IV) based on lung function testing (post-bronchodilator FEV_1_/FVC < 70%) and a prescribed permanent inhaled therapy. Inhaled therapy was prescribed according to the risk assessment (A – D) as proposed in the GOLD report 2011 [24].

All patients discharged in 2012 were screened looking for a diagnosis with ICD-10-Code 44.0–44.9 at time of discharge. COPD diagnosis and stage were verified by the most recent lung function performed in 2012. Based on lung function criteria, patients with partial post-bronchodilator reversibility were included. However, patients with complete reversibility (∆FEV_1_ > 12%, or > 0.2 l) were excluded.

Adherence to inhaled therapy, based on the 24 months observation period, was defined according to the percentage of prescribed inhalers dispensed to the patient and classified as follows: Complete adherence (> 80%), partial adherence (50–80%) and low adherence (< 50%). 80% is a frequently used threshold for the differentiation of adherence (high or low) [23]. We decided to further divide the participants according to adherence into three groups to show more precise results in the low adherence group (partial and low adherence).

Additionally, adherence was reported as mean medication possession ratio (MPR) [25], and categorized by sex, FEV_1_%pred, smoking status and inhaled therapy. The MPR was calculated using the ratio of personal adherence months to the whole observation period of each participant.

For a permanent inhaled therapy, one medical prescription per month for each device was assumed for complete adherence. Complete data therefore was provided by the Upper Austrian Health Insurance (OÖGKK).

Statistical analysis was performed using SAS 9.3. Figures and tables were created with Microsoft Excel 2016. The adherence category (complete, partial, low) was the underlying and central variable in all statistical analyses performed in this study. The association between adherence and most important covariates (age, sex, FEV_1_%pred, smoking status) is shown in a descriptive overview using means and proportions.

Nonparametric Chi-square test, Mann-Whitney U test and t-test were used to investigate differences between groups according to adherence category.

COPD control was assessed using the rate of severe exacerbations leading to hospitalization per year. Concerning exacerbations leading to hospitalization a binary univariate and multivariate logistic regression analysis was performed, based on odds ratios, to determine the influence of several factors (age, sex, FEV_1_%pred, smoking status, adherence) on exacerbation rates.

Results are mainly expressed as frequencies or as mean. Statistical significance was defined as *p* <  0.05 for all analyses in this study.

## Results

**Fig. 1:**
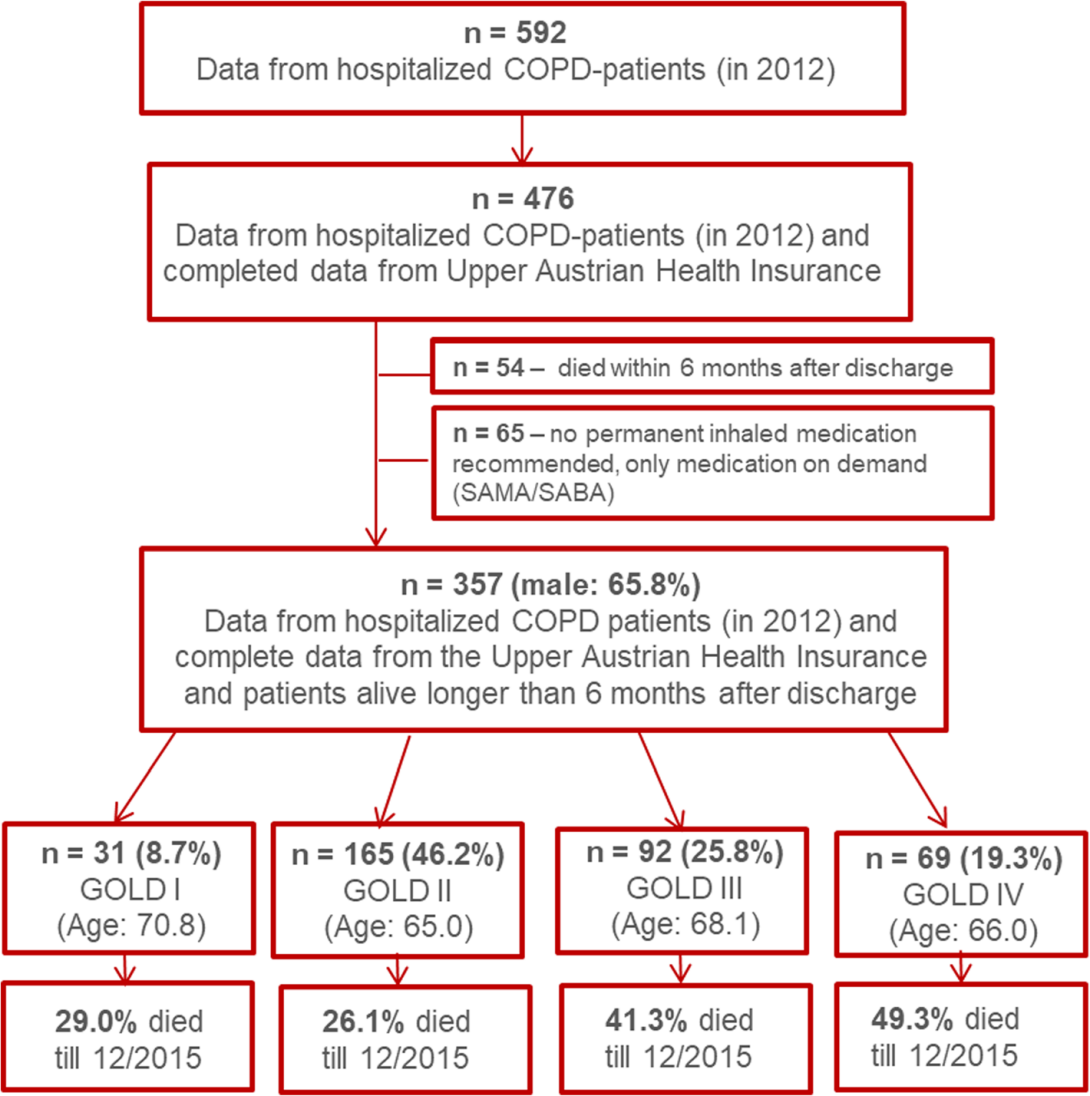
Study population

Out of 592 hospitalized patients with COPD and discharged with a guideline conform inhaled therapy in 2012, complete data was available of 476 cases in the database of the Upper Austrian Health Insurance (OÖGKK). 54 subjects died within 6 months after discharge and 65 had no prescription for permanent inhaled medication and were therefore excluded (Fig. 1).

Out of 357 patients, 65.8% were male and 34.2% were female, with a mean age of 66.5 years and a mean FEV_1_ of 55.0%pred. 55% had GOLD spirometry class I – II COPD and 45% had GOLD spirometry class III – IV COPD. Overall, 35.3% were current smokers, 57.4% former smokers and only 3.9% were never smokers. In 77.0% of all cases, inhaled triple therapy (LAMA + LABA + ICS) was prescribed at the time of discharge (for other inhaled therapies see Table 1). 74.2% had an additional prescription for SAMA and/or SABA, as inhaled therapy on demand. 17% of all subjects were on long-term oxygen therapy. There was no significant difference between men and women concerning age, FEV_1_, smoking status or long-term oxygen therapy. However, significantly more male patients were treated with triple therapy (83.0% vs 65.6%; *p* <  0.001).

**Table 1:** Characteristics of the participants at baseline

	All	Female	Male	*p*-value
n (%)	357	122 (34.2)	235 (65.8)	
Age in year, mean (SD)	66.5 (10.6)	66.1 (11.1)	66.7 (10.0)	0.580
FEV_1_%pred., mean (SD)	55.0 (18.5)	58.0 (19.4)	53.4 (17.9)	0.067
Smoking status, n (%)				
Current smoker	126 (35.3)	43 (35.3)	83 (35.3)	0.982
Former smoker	205 (57.4)	67 (54.9)	138 (58.7)	0.471
Never smoker	14 (3.9)	8 (6.5)	6 (2.6)	0.065
No information	12 (3.4)	4 (3.3)	8 (3.4)	
Inhaled Therapy, n (%)				
LAMA only	10 (2.8)	3 (2.5)	7 (3.0)	0.777
LABA only	3 (0.8)	2 (1.6)	1 (0.4)	0.233
LABA + ICS	64 (17.9)	34 (27.9)	30 (12.8)	< 0.001
LAMA + LABA + ICS	275 (77.0)	80 (65.6)	195 (83.0)	< 0.001
LABA + LAMA	5 (1.4)	3 (2.5)	2 (0.9)	0.952
LTOT, n (%)	60 (17.0)	26 (21.5)	34 (14.7)	0.101

LAMA – long-acting muscarinic antagonist; LABA – long-acting beta-adrenoceptor agonist; ICS – inhaled corticosteroid; LTOT – long-term oxygen therapy

33.6% of 357 patients showed complete adherence to their therapy (33.2% in men, 34.4% in women), with a mean age of 67.0 years and a mean FEV_1_ of 49.2% predSubjects with complete adherence to therapy had a significantly lower FEV_1_ compared to those with low adherence (49.2%pred. vs 59.2%pred., respectively; p <  0.001). (for further baseline characteristics by adherence see Table 2).

**Table 2:** Baseline characteristics of patients by adherence; n = 357

	Complete adherence (> 80%)	Partial adherence (50–80%)	Low adherence (< 50%)	All	*p*-value
n (%)	120 (33.6)	85 (23.8)	152 (42.6)	357	
Sex, n (%)					0.865
Female (%, *n* = 122)	42 (34.4)	27 (22.1)	53 (43.5)	122	
Male (%, *n* = 235)	78 (33.2)	58 (24.7)	99 (42.1)	235	
Age in years, mean (SD)	67.0 (9.2)	66.7 (9.9)	66.0 (11.5)	66.5 (10.4)	0.920
FEV_1_%pred, mean (SD)	49.2 (17.6)	56.0 (18.3)	59.2 (18.2)	55.0 (18.5)	< 0.001
Smoking status, n (%)					0.081
Current smoker	33 (27.5)	31 (36.5)	62 (40.8)	126 (35.3)	
Former smoker	81 (67.5)	48 (56.5)	76 (50.0)	205 (57.4)	
Never smoker	3 (2.5)	3 (3.5)	8 (5.3)	14 (3.9)	
No information	3 (2.5)	3 (3.5)	6 (3.9)	12 (3.4)	

**Fig. 2:**
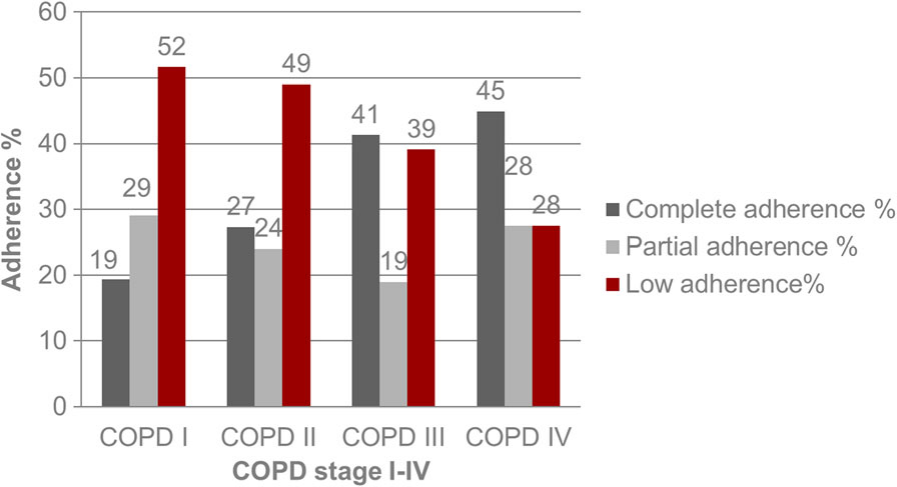
Adherence by stage of COPD, *n* = 357

Among all 357 patients, complete adherence was noted in 44.9% of GOLD spirometry category IV participants, while only 19.4% of GOLD spirometry category I were noted to be completely adherent (Fig. 2).

Using the medication possession ratio (MPR), to describe adherence, the overall mean MPR was 0.565. Hence, on average patients were adherent in 56.5% of all months during the observation period.

Male patient had slightly higher MPRs than females (0.568 vs 0.558, respectively; *p* = 0.883). MPRs by GOLD spirometry class I – IV were 0.486, 0.534, 0.609 and 0.755, respectively. These differences were statistically highly significant (*p* = 0.002). Former smokers had a higher mean MPR (0.610) compared to smokers (0.510) and never smokers (0.464) (*p* = 0.021). Patients on triple therapy (LABA + LAMA + ICS) were, statistically not significant, more adherent compared to patients on other therapy regimes (0.584 vs 0.499, respectively; *p* = 0.089).

Table 3 shows the results of a binary univariate and multivariate logistic regression analysis of factors independently associated with exacerbations leading to hospitalizations during the observation period. The risk of exacerbations leading to hospitalization was 10-fold higher in GOLD stage IV compared to GOLD stage I (OR 10.69; CI 2.99; 38.24) in univariate analysis. In the univariate and multivariate analysis, an inverse association between adherence to therapy and exacerbations leading to hospitalization was observed. Subjects with low adherence had a reduced risk of exacerbations (not significant in multivariate analysis; OR 0.58; 95% CI (0.33; 1.02)); for details see Table 3).

**Table 3:** Parameters independently associated with severe exacerbations/ hospitalizations; n = 357

	OR (95% CI) crude	OR (95% CI) adjusted
Gender		
Female	Reference	Reference
Male	0.89 (0.58; 1.38)	0.95 (0.57; 1.57)
Age (years)		
< 50	Reference	Reference
50–60	1.68 (0.56; 5.06)	0.87 (0.26; 2.90)
60–70	2.12 (0.74; 6.07)	1.00 (0.32; 3.13)
> 70	1.12 (0.39; 3.21)	0.71 (0.22; 2.24)
FEV_1_		
> 80% pred.	Reference	Reference
50–80% pred.	0.86 (0.39; 1.88)	0.94 (0.40; 2.24)
30–50% pred.	2.09 (0.92; 4.75)	2.50 (1.02; 6.13)
< 30% pred.	10.69 (2.99;38.24)	13.62 (3.11; 59.63)
Smoking status		
Current smoker	Reference	Reference
Former smoker	1.11 (0.71; 1.73)	1.19 (0.70; 2.02)
Never smoker	0.67 (0.21; 2.12)	0.82 (0.23; 2.92)
Adherence to therapy		
Complete (≥ 80%)	Reference	Reference
Partial (50–80%)	0.77 (0.44; 1.35)	0.95 (0.50; 1.78)
Low (< 50%)	0.44 (0.27; 0.71)	0.58 (0.33; 1.02)

## Discussion

In our retrospective data analysis, we were able to show that adherence to inhaled therapy in COPD patients is generally low. Complete adherence to inhaled therapy was only seen in 33.6%. Factors associated with better adherence were age, former smoking, and more severe airflow limitation.

In prior studies adherence in COPD ranged between 70 to 90% in several clinical trials; however, in clinical practice, adherence is lower within the range of 10–40%, irrespective of the probable insufficient or incorrect use of the device [15, 26, 27].

We could show that adherence to inhaled therapy was higher in GOLD spirometry class III – IV COPD and was highest in patients with GOLD spirometry class IV COPD. This may be due to the fact, that with advanced disease and a higher burden of symptoms, the inhaled medication is perceived more necessary by the patient. The association between symptom relief and medication use may be a potent trigger for better adherence [16]. Contrariwise, lack of clinical symptoms can be misinterpreted and can lead to treatment interruption and cessation [26]. This is in accordance with previous studies, where adherence was better in patients with more severe disease [28, 29].

In our univariate and multivariate analysis, the risk of exacerbations leading to hospitalization was more than 10-fold higher in GOLD stage IV compared to GOLD stage I. Patients with low adherence tended to have a reduced risk for exacerbations leading to hospitalization (OR 0.58; 0.33, 1.02; not significant in multivariate analysis).

In previous research, better adherence in COPD patients was associated with a reduced risk for exacerbations and health care utilization [18, 30].

This paradoxical result may be caused by other influencing factors as this trend was considerably less pronounced and statistically not significant in multivariate compared to univariate analysis. Furthermore, the nonadherent patients predominately had GOLD spirometry class I – II COPD with less impairment of lung function, probably less symptoms and better quality of life.

Adherence in COPD patients is complex and multiple factors may be influencing. Parameters associated with poor adherence include the dosing regime, drug side effects, comorbidities, age and costs, the patient’s disease perception but also social factors [14, 15, 23].

Possibilities to improve adherence include knowledge about self-management, overcoming misperceptions, close communication and shared decision-making between patients and their physicians, simple therapy regimes and low out-of-pocket costs for medications [13, 18, 23, 26, 31–33].

### Limitations

Due to administrative limitations, we could only include patients insured by the Upper Austrian Health Insurance (OÖGKK). Therefore, we have no data for subjects insured by other health insurance companies. However, the Upper Austrian Health Insurance covers more than 80% of the general Upper Austrian population.

As a noninterventional study, we were not able to use electronically monitored inhalers, adherence scores or questionnaires to evaluate adherence. Although prescription refill rates are widely used in scientific literature, they may not perfectly reflect the electronically monitored inhaler use of COPD patients [34]. Moreover, refill rates might depend on social support and cognitive abilities of COPD patients [35–37]. Unfortunately, there is no data about the social framework of our patients available. As adherence was classified by dispensed inhalers, we are not able to evaluate if the patients have taken their medication correctly.

## Conclusion

Complete adherence to inhaled therapy was only seen in about one third of subjects with a prior hospitalization due to a COPD exacerbation. Adherence was significantly higher among those with spirometrically more severe COPD.

Identifying reasons for and a better understanding of underlying causes of poor adherence are necessary and warrant further research.

## Abbreviations

COPD: chronic obstructive pulmonary disease
FEV_1_: forced expiratory volume in 1 s
FVC: forced vital capacity
GOLD: Global Initiative for Chronic Obstructive Lung Disease
ICD-10: 10th revision of the International Statistical Classification of Diseases
ICS: inhaled corticosteroid
LABA: long-acting beta-adrenoceptor agonist
LAMA: long-acting muscarinic antagonist
MPR: medication possession ratio
pred.: predicted
SABA: short-acting beta-adrenergic agonist
SAMA: short-acting muscarinic antagonist
